# Overexpression of Toll-Like Receptor 4 Affects Autophagy, Oxidative Stress, and Inflammatory Responses in Monocytes of Transgenic Sheep

**DOI:** 10.3389/fcell.2020.00248

**Published:** 2020-05-05

**Authors:** Sutian Wang, Xuting Song, Kunli Zhang, Shoulong Deng, Peixin Jiao, Meiyu Qi, Zhengxing Lian, Yuchang Yao

**Affiliations:** ^1^College of Animal Science and Technology, Northeast Agricultural University, Harbin, China; ^2^State Key Laboratory of Livestock and Poultry Breeding, Guangdong Key Laboratory of Animal Breeding and Nutrition, Institute of Animal Science, Guangdong Academy of Agricultural Sciences, Guangzhou, China; ^3^Institute of Animal Health, Guangdong Academy of Agricultural Sciences, Key Laboratory of Livestock Disease Prevention of Guangdong Province, Scientific Observation and Experiment Station of Veterinary Drugs and Diagnostic Techniques of Guangdong Province, Ministry of Agriculture and Rural Affairs, Guangzhou, China; ^4^Chinese Academy of Sciences (CAS) Key Laboratory of Genome Sciences and Information, Beijing Institute of Genomics, Chinese Academy of Sciences, Beijing, China; ^5^Institute of Animal Husbandry, Heilongjiang Academy of Agricultural Sciences, Harbin, China; ^6^Beijing Key Laboratory for Animal Genetic Improvement, National Engineering Laboratory for Animal Breeding, Key Laboratory of Animal Genetics and Breeding of the Ministry of Agriculture, College of Animal Science and Technology, China Agricultural University, Beijing, China

**Keywords:** TLR4, autophagy, oxidative stress, inflammatory responses, transgenic animal model, p38 MAPK

## Abstract

Toll-like receptor 4 (TLR4) is a critical pattern recognition receptor that plays a critical role in the host innate immune system’s recognition of Gram-negative bacteria. Since it is the lipopolysaccharide (LPS) receptor, it links the activated inflammatory response with autophagy and oxidative stress. Autophagy, or type II programmed cell death, was reported to have defensive functions in response to the production of inflammatory cytokines and oxidative stress. To explore the relationship between autophagy, inflammation, and oxidative stress, a TLR4-enriched transgenic (Tg) animal model (sheep) was generated. Autophagy activity in the Tg blood monocytes was significantly higher than in the wild-type animal under LPS stress, and it returned to normal after transfection of TLR4 siRNA. Pretreatment with 3-methyladenine (3-MA) inhibited autophagy and enhanced oxidative stress and the production of TNF-α. The LPS-induced reactive oxygen species (ROS) level was markedly increased in the Tg group at an early stage before quickly returning to normal values. In addition, suppressing ROS production by *N*-acetyl-L-cysteine down-regulated the number of intracellular autophagosomes and the expression of Beclin-1, ATG5, and cytokines IL-1β, IL-6, and TNF-α. Further mechanistic investigation suggested that the TLR4-associated p38 mitogen-activated protein kinase (MAPK) signaling pathway was involved in autophagy and oxidative stress. P38 MAPK promotes intracellular autophagy, ROS production, and inflammatory response. Moreover, TLR4 over-expression suppressed oxidative stress and the production of inflammatory cytokines and increased autophagy activity *in vivo*. Taken together, our results showed that LPS induced autophagy, which was related to TLR4-mediated ROS production through the p38 MAPK signaling pathway. In addition, our study also provided a novel transgenic animal model to analyze the effects of TLR4 on autophagy, oxidative stress, and inflammatory responses.

## Introduction

Pattern recognition receptors (PRRs) play important roles in the recognition of microbial molecules by the animal’s innate immunity (Janeway and Medzhitov, 2002). These molecules, termed pathogen-associated molecular patterns (PAMPs), can activate multiple inflammatory signaling pathways to clear the infection (Takeuchi and Akira, 2010). Toll-like receptors (TLRs) are PRRs found mainly on membranes (Chen et al., 2009). An essential element of this family is Toll-like receptor 4 (TLR4), which mainly recognizes lipopolysaccharide (LPS), one of the inflammatory PAMPs that exist in the cell walls of most Gram-negative bacteria. After the host has been infected with these bacteria, multiple oxidation intermediates and inflammatory cytokines can be activated through TLR4 (Ryan et al., 2004). TLR4 interacts with other proteins to form a TLR4/MD2/CD14 complex that can bind to LPS (Hoareau et al., 2010). Then, TLR4 recruits adaptors to trigger myeloid differentiation primary response gene 88 (MyD88)-dependent and MyD88-independent signaling (Tse et al., 2014), ultimately leading to the production of reactive oxygen species (ROS) and inflammatory responses.

Generally, ROS is maintained at a certain level to induce an oxidative reaction that helps to clear pathogenic bacteria, but an excess of oxidative stress will cause damage to the host. It has been shown that LPS-generated ROS and nicotinamide adenine dinucleotide phosphate oxidase (NOX) are closely related to ROS (Park et al., 2006; Yu et al., 2015). NOX1, NOX3, NOX4, NOX 5, DUOX1, and DUOX2 are collectively known as the NOX family proteins, which mainly exist in phagocytes and can produce ROS through the transfer of electrons. TLR4 downstream signaling, namely, the interleukin-1 receptor-associated kinase-4 (IRAK-4), is involved in controlling NOX (Pacquelet et al., 2007). However, some research suggested that the TLR4-NOX2/4 signaling pathway influences the activated state of autophagy and inflammatory factors (Suzuki et al., 2012; Sciarretta et al., 2014). Thus, ROS may be involved in the communication between TLR4 and autophagy.

Essentially, autophagy is a lysosomal degradation process that can eliminate damaged organelles and pathogenic bacteria to maintain cell homeostasis and survival (Singh and Cuervo, 2011). It has been reported that the antioxidant reaction and autophagy that were induced under oxidative stress helped to concomitantly down-regulate ROS levels, inflammatory responses, and oxidative damage to organelles (Kim et al., 2017). It must be emphasized that this repair system enables the cell to reach a new homeostasis. Although these studies suggested a close relationship between ROS and autophagy, the interconnectedness between TLR4, ROS, the inflammatory response, and autophagy remains to be addressed in detail, which was the goal of this study.

TLR4 participates in the innate immunity response by mainly sensing LPS and then activating a variety of antimicrobial immune responses. Our previous studies have shown that, compared to the control cells, the peripheral blood monocytes from Tg sheep overexpressing TLR4 evoked a stronger inflammatory response under LPS stress at an early stage and dropped quickly back to initial levels (Deng et al., 2012). In addition, the increased NO secretion induced a stronger intracellular oxidative stress that was due to the overexpression of TLR4 (Deng et al., 2015). Furthermore, the overexpression of TLR4 contributed to the phagocytosis of pathogenic bacteria *via* the p38 mitogen-activated protein kinase (MAPK) and phosphatidylinositide3-kinase (PI3K) signaling pathways (Wang et al., 2018a, b). Here we continued this line of investigation by first measuring the effects of TLR4 (overexpression and inhibition) on the interactions between oxidative stress and autophagy. Then, the inflammatory responses during TLR4-mediated oxidative reaction and autophagy were assessed. Finally, the antioxidant NAC and autophagy inhibitor 3-methyladenine (3-MA) were used to analyze the deep molecular mechanisms under the TLR4-mediated LPS stress. We present the first investigation of the interconnectedness between TLR4, ROS, inflammatory response, and autophagy in a Tg model overexpressing TLR4.

## Materials and Methods

### Animal Ethics Statement

All the animal experiments and treatments followed the guidelines of the Animal Welfare Committee of the Northeast Agricultural University, and all the experiments were approved by the Animal Welfare Committee of the Northeast Agricultural University.

### Production and Detection of Tg Sheep

**FIGURE 1:**
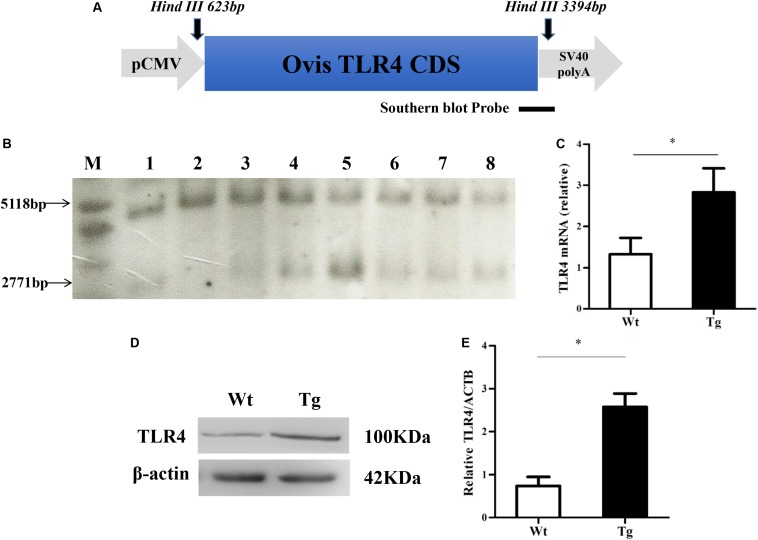
Southern blot and Western blot analysis of Tg sheep. **(A)** Construction of the CMV-Ovis TLR4 expression vector. **(B)** Southern blot analysis of partial Tg sheep. The endogenous TLR4 locus has a 5,118 bp signature band, and the transgene produces a 2,771 bp band. M, marker (1 kb ladder); 1–8, eight sheep: the wild sheep is 2 and the Tg sheep are 1, 3, 4, 5, 6, 7, and 8. **(C)** Quantitative real time PCR analysis of TLR4 expression level. **(D,E)** The protein level of TLR4. Wt, wild-type sheep; Tg, transgenic sheep. All data are presented as the mean ± SEM from three experiments. ^∗^*P* < 0.05 vs. Wt group.

Tg sheep were produced by transferring the linearized vector (digested with *Ase*I) into the pronuclei of fertilized eggs by microinjection (Figure 1A). Generally, the transformed exogenous TLR4 genes were detected by Southern blot. The genomic DNA was extracted from peripheral blood monocytes. *Hin*dIII (NEB) was used to digest genomic DNA. The probe was generated by PCR with the following primer pair: forward (F), 5′-ACTGGTAAAGAACTTGGAGGAGG-3′ and reverse (R), 5′-CCTTCACAGCATTCAACAGACC-3′, producing a 671-bp amplicon that was labeled by digoxigenin (Roche). The total RNA of peripheral blood monocytes was extracted with TRIzol reagent (Invitrogen), and the PrimeScript RT Reagent Kit (TAKARA) was used to prepare the cDNA. The expression of TLR4 mRNA was measured by quantitative real-time PCR (qRT-PCR) *via* the ABI 7500 system with SYBR Premix Ex Taq II kit (TAKARA) according to the instructions. β-Actin was chosen to normalize the data of each sample. The TLR4 and β-actin primer sequences were as follows: TLR4, (F) 5′-ATCATCAGCGTGTCGGTTGTCA-3′ and (R) 5′-GCAGCCAGCAAGAAGCATCAG-3′; β-actin, (F) 5′-AGATGTGGATCAGCAAGCAG-3′ and (R) 5′-CCAATCTCATCTCGTTTTCTG-3′. The relative expression of mRNA was calculated by the 2^–ΔΔCT^ method.

### Sheep Peripheral Blood Monocyte Isolation and Culture

Sheep were divided into two groups: Tg sheep and wild-type (WT) sheep (*n* = 3 in each group). Sheep peripheral blood monocytes were isolated from the blood of sheep using the separation medium (Tbdscience). The cells were incubated at 37°C in a 5%-CO_2_ incubator for 2 h and then the non-adherent cells were washed out. The adherent cells were cultivated in RPMI1640 (Gibco) containing 10% fetal bovine serum (Gibco) at 37°C in a 5%-CO_2_ incubator.

### Western Blotting

The cells were harvested and lysed using RIPA buffer (Beyotime) with protease inhibitor cocktail and PMSF (Roche). Then, the proteins were quantified using the BCA Protein Assay Kit (Thermo Fisher Scientific). Equal amounts of proteins were resolved on 12% SDS-PAGE and transferred to a polyvinylidene fluoride membrane (Millipore). After incubation with primary antibodies against TLR4 (1:1,000; Affinity, AF7017), LC3B (1:1,000; Abcam, ab51520), ATG5 (1:1,000; Sigma, A0856), Beclin-1 (1:1,000; CST, 3495), actin (1:5,000; CST, 4970), GAPDH (1:5,000; Proteintech, 10494-1-AP), and horseradish peroxidase-conjugated secondary antibodies (1:1,000; Beyotime, A0208), the membranes were visualized by enhanced chemiluminescence (Thermo Fisher Scientific). The protein bands were analyzed by ImageJ software (National Institutes of Health; version 1.45).

### Transfection of Small Interfering RNA

To knock-down the expression of TLR4, sheep peripheral blood monocytes were transfected with siRNA-specific TLR4 from Genepharma (si-TLR4-86: sense, 5′-GCGU ACAGGUUGUUCCUAATT-3′ and antisense, 5′-UUAGGAAC AACCUGUACGCTT-3′). Transfection was accomplished with lipofectamine RNAiMAX (Invitrogen) according to the manufacturer’s instructions.

### Transmission Electron Microscopy

The monocytes were treated with LPS (100 ng/ml) (Sigma, L6529) for 12 h, and then the cells were collected to measure the autophagy level by transmission electron microscopy. In inhibitory experiments, the cells were pretreated with 10 mM 3-MA for 6 h, and LPS (100 ng/ml) was added for another 12 h. Briefly, the monocytes were collected and fixed in 2.5 glutaraldehyde for 24 h and then in 1% osmic acid for 1 h. The cells were dehydrated in a graded series of ethanol and embedded in epoxyresin. Ultrathin sections were observed under an H-7650 microscope at 100 kV (Hitachi).

### Laser Scanning Confocal Microscopy

The monocytes were treated with LPS (100 ng/ml) (Sigma, L6529) for 12 h, and then the cells were collected to measure the autophagy level by transmission electron microscopy. In inhibitory experiments, the cells were pretreated with 10 mM 3-MA for 6 h, and LPS (100 ng/ml) was added for another 12 h. Then, the cells were fixed in 4% paraformaldehyde for 20 min. The fixed cells were permeabilized by 0.3% Triton X-100 for 10 min. The cells were incubated with primary antibodies against LC3B (1:1,000, Abcam, ab51520) at 4°C for 12 h and then incubated with Cy3-labeled secondary antibody (1:500; Beyotime, A0516) and hoechst33342 (Solarbio). The level of LC3B was visualized using a laser scanning confocal microscope (Leica SP8) and a × 63 oil objective.

### Detection of Intracellular ROS

LPS-induced intracellular ROS was measured by adding dichlorodihydrofluorescein diacetate (DCFH-DA) at different time points (0, 2, 8, and 24 h). Intracellular O^2–^ and H_2_O_2_ production was monitored by testing the fluorescence caused by oxidative DCF, and the green fluorescence signal indicates the ROS level. After the cells were treated with LPS, 10 μM of DCFH-DA was added to the cells for 30 min at 37°C. The intracellular ROS level was visualized using a fluorescence microscope (Olympus, X71).

### Measurement of Oxidative Stress

The monocytes were stimulated with LPS for 12 h, and the culture supernatants were collected with a detection kit (Njjcbio) according to the manufacturer’s instructions to examine the contents of NO and malondialdehyde (MDA) by spectrophotometry.

### Determination of the Signals of Autophagy and ROS in Monocytes

To analyze the interactions between TLR4, autophagy, and ROS, several inhibitors were used, namely, Tak242 – a TLR4 inhibitor (Sigma, pretreated for 6 h), 3-MA – an autophagy inhibitor (Sigma, pretreated for 6 h), NAC – an antioxidant and a free radical scavenger (Sigma, pretreated for 2 h), and SB203580 – a specific inhibitor of p38 MAPK (Sigma, pretreated for 1 h). After treating the cells with these inhibitors, the cells were harvested for detection of autophagy and oxidative level, and the supernatants were collected to test the inflammatory cytokines.

### ELISA Assay for Inflammatory Cytokines

The monocytes were treated with LPS (100 ng/ml) for 12 h, and then the cell culture supernatants were collected to measure the protein concentrations of IL-1β, IL-6, IL-12, and TNF-α using ELISA kits (CUSABIO) according to the manufacturer’s instructions.

### Injection of Lipopolysaccharide *In vivo*

Six-month-old transgenic sheep and wild-type sheep (*n* = 3 in each group) were subcutaneously injected with LPS (0.04 mg/ml and 0.25 μg/kg). At 0, 2, and 12 h after the LPS challenge, the serum was isolated for tests of proinflammatory cytokines and oxidative stress. Peripheral blood mononuclear cells (PBMCs) were collected for qRT-PCR. In qRT-PCR tests, GAPDH was chosen to normalize the data of each sample. Beclin-1, ATG5, WIPI1, and GAPDH primer sequences were as follows: Beclin-1: (F) 5′-GTCACCATCCAGGAGCTCACA-3′ and (R) 5′-CACCATCCTGGCGAGTTTCA-3′; ATG5: (F) 5′-GCTTTAC TCCACTGCCGTCA-3′ and (R) 5′-ACCAATGTTTCCAC TCCCTC-3′; WIPI1: (F) 5′-TCAGGAACCAGCGAAGAGA-3′ and (R) 5′-ACGGCACGAAGTGATAGGA-3′; and GAPDH: (F) 5′-GTGTCTGTTGTGGATCTGACCTG-3′ and (R) 5′-AGAAGAGTGAGTGTCGCTGTTGAAGT-3′. The relative expression of mRNA was calculated by the 2^–ΔΔCT^ method.

### Statistical Analysis

All data are shown as mean ± SEM, and individual experiments were repeated not fewer than three times. Statistical analyses were performed by the univariate analysis of variance (ANOVA) followed by Student’s *t*-test. *P* < 0.05 was considered to be statistically significant.

## Results

### Screening of Tg Sheep Overexpressing TLR4

The Ovis TLR4 CDS region was first cloned and linked to an expression vector that has a pCMV promoter (Figure 1A). Then, the linearized vector (digested with *Ase*I) was transferred into the pronuclei of fertilized eggs by microinjection. The use of the restriction enzyme, *Hin*dIII, resulted in a 5,118 bp endogenous fragment present in all individual sheep and a 2,771 bp exogenous fragment that only exists in Tg sheep (Figure 1B). The relative TLR4 expression level in monocytes derived from peripheral blood was detected by qRT-PCR. The result indicated that the expression of TLR4 was significantly increased compared to the Wt control (^∗^*P* < 0.05) (Figure 1C). The Ovis TLR4 protein level in Tg sheep was also higher than that in Wt control (^∗^*P* < 0.05) (Figures 1D,E).

### TLR4 Overexpression Increases Autophagy Activity

**FIGURE 2:**
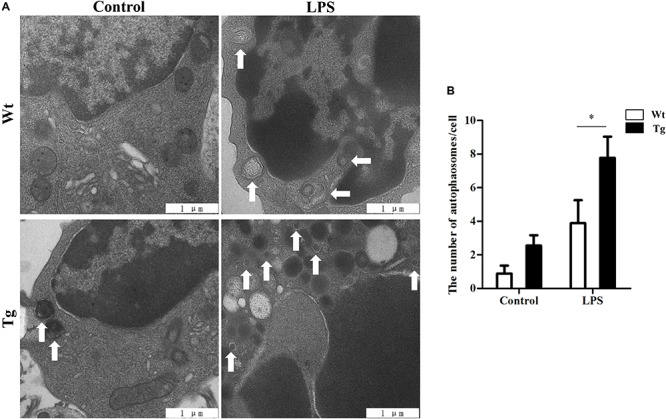
Autophagosomes/autolysosomes increase in Tg sheep. **(A)** Transmission electron microscopy images: the white arrows indicate autophagosomes/autolysosomes; ×20,000; scale bar: 1 μm. **(B)** Statistical analysis of the number of autophagosomes/autolysosomes per cell. Statistical analysis of not fewer than 25 cells. Wt, wild-type sheep; Tg, transgenic sheep. All data are presented as the mean ± SEM from three experiments. ^∗^*P* < 0.05 vs. Wt group.

To examine the autophagy activity in sheep peripheral blood monocytes, the autophagosomes were detected under transmission electron microscopy (TEM) (Figure 2). The autolysosomes in the TLR4-overexpression group were increased compared to those of the Wt group. After stimulating the cells with LPS (100 ng/ml for 12 h), the number of autophagosomes in the Tg group was significantly higher than that in the Wt group (^∗^*P* < 0.05).

**FIGURE 3:**
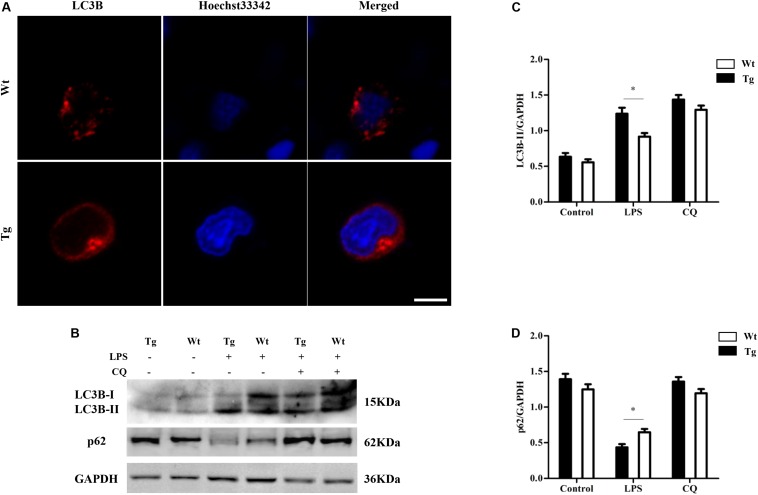
LC3B increases in Tg sheep. **(A)** Immunofluorescence images of LC3B under laser scanning confocal microscope. Red: Cy3-labeled LC3B protein; blue, Hoechst33342-labeled nuclei; scale bar: 5 μm. **(B–D)** Analysis of LC3B and p62 level by Western blot. Wt, wild-type sheep; Tg, transgenic sheep. ^∗^*P* < 0.05 vs. Wt group.

To further examine autophagy activity, the protein levels of the autophagosomal marker LC3B were detected by laser scanning confocal microscopy. LC3B protein was tagged by Cy3 (red), and the nuclei were tagged by hoechst33342 (blue). After stimulating the cells with LPS (100 ng/ml, for 12 h), the images showed a distinctly increasing level of LC3B in the Tg group compared to the Wt group (^∗^*P* < 0.05) (scale bar: 5 μm; Figure 3A). In order to further determine whether the overexpression of TLR4 promotes the monocyte autophagy activity, p62 levels were also detected. LC3-II levels were up-regulated with LPS treatment and p62 levels were down-regulated. In addition, LPS-induced down-regulation of p62 could be reversed by chloroquine (CQ) treatment. After stimulating the cells with LPS, the results also showed an increasing level of LC3-II in the Tg group compared to the Wt group; meanwhile, the p62 levels in the Tg group was lower than that in the Wt group (Figures 3B–D). These results suggest that TLR4-overexpression increases the autophagy activity in sheep peripheral blood monocytes.

### Knock-Down of Ovis TLR4 Inhibits Autophagy Activity

**FIGURE 4:**
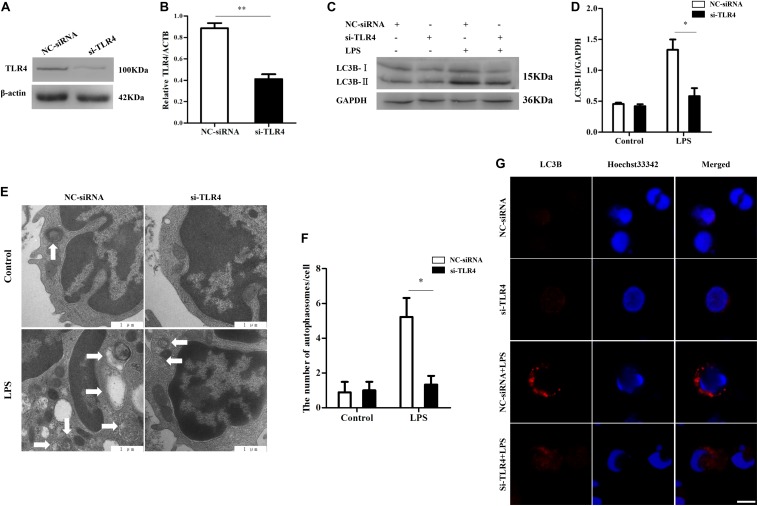
Inhibition of TLR4 by RNAi decreases autophagy activity. **(A,B)** Western blot analysis of the TLR4 protein levels after TLR4 siRNA transfection. **(C,D)** Analysis of LC3B levels with RNAi treatment by Western blot. **(E)** Transmission electron microscopy images of autophagosomes/autolysosomes; ×20,000 scale bar: 1 μm. **(F)** Analysis of the number of autophagosomes/autolysosomes per cell. Statistical analysis of not fewer than 25 cells. **(G)** Confocal microscopy image of LC3B levels in monocytes. Red: LC3B protein; blue, Hoechst33342-labeled nuclei; scale bar: 5 μm. NC-siRNA, negative control siRNA; si-TLR4, TLR4 siRNA. ^∗^*P* < 0.05 vs. si-TLR4 group, **P < 0.01 vs si-TLR4 group.

To further investigate the function of TLR4 during autophagy, RNAi was used to inhibit the expression of TLR4 in monocytes of sheep. Western blot results showed that the transfection of monocytes with si-TLR4 notably reduced the TLR4 protein level (^∗∗^*P* < 0.01) (Figures 4A,B). Furthermore, the down-regulation of TLR4 by siRNA decreased the autophagosomes/autolysosomes and LC3B-II protein compared to the negative control siRNA (NC-siRNA) group (^∗^*P* < 0.05) (Figures 4C–F). In addition, immunofluorescence staining showed that the LC3B level was significantly lower in the si-TLR4 group than in the NC-siRNA group (^∗^*P* < 0.05) (Figure 4G). These results suggest that the down-regulation of TLR4 leads to a decrease in LPS-induced autophagy activity.

### Ovis TLR4 Effects on ROS Production

**FIGURE 5:**
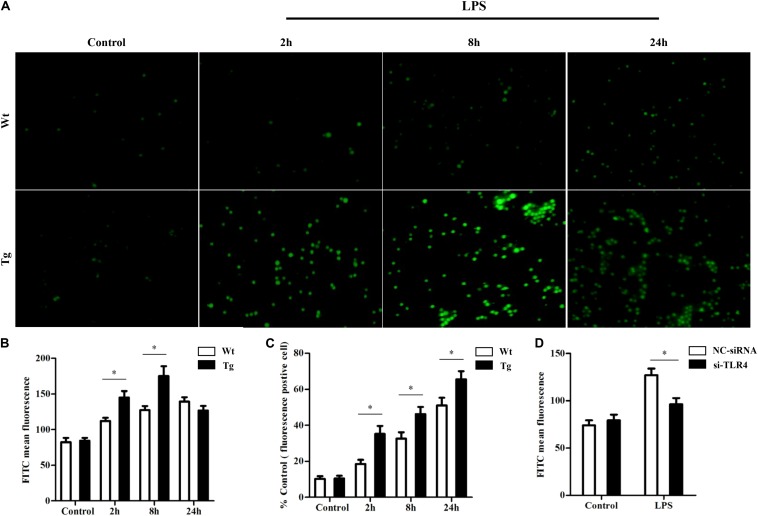
TLR4 effects on reactive oxygen species (ROS) production by TLR4 overexpression or RNAi. **(A,B)** Analysis of ROS by DCFH-DA in monocytes. ROS was detected by dichlorodihydrofluorescein diacetate, and a fluorescence microscope was used to count the positive cells, ×200. **(C)** ROS fluorescent positive cells; statistical analysis of not fewer than 100 cells. ^∗^*P* < 0.05 vs. Wt group. **(D)** TLR4 RNAi effects on ROS production. ^∗^*P* < 0.05 vs. NC-siRNA group.

As some studies have suggested that ROS production could induce autophagy and the above results showed that TLR4 influenced autophagy activity, we proceeded to explore whether TLR4 could affect ROS production. DCFH-DA staining showed that the overexpression of TLR4 promoted intracellular ROS production at an early stage (2–8 h), and then the ROS level quickly returned to normal values under LPS stress (Figures 5A–C). Furthermore, inhibition of TLR4 by si-TLR4 decreased the LPS-induced ROS (Figure 5D). These results suggest that TLR4 regulated the LPS-induced ROS production in sheep monocytes.

### TLR4 Activates the P38 MAPK Signaling Pathway and ROS Production to Promote LPS-Induced Autophagy

**FIGURE 6:**
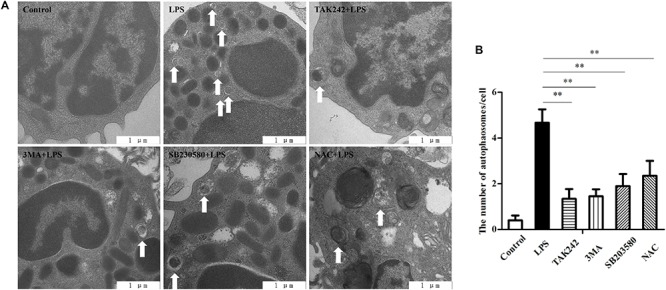
TLR4-associated signaling is involved in the formation of autophagosomes/autolysosomes. **(A)** Cells were pretreated with either DMSO (0.1%) or TAK242 (30 μM) for 6 h or 3MA (10 mM) for 6 h, SB203580 (5 μM) for 1 h, or NAC (1 mM) for 2 h, and then the cells were treated with LPS (100 ng/ml) for another 6 h. Transmission electron microscopy was used to survey the autophagy, ×20,000; scale bar: 1 μm. **(B)** Statistical analysis of the number of autophagosomes per cell. Statistical analysis of not fewer than 25 cells. ^∗∗^*P* < 0.01 vs. LPS group.

**FIGURE 7:**
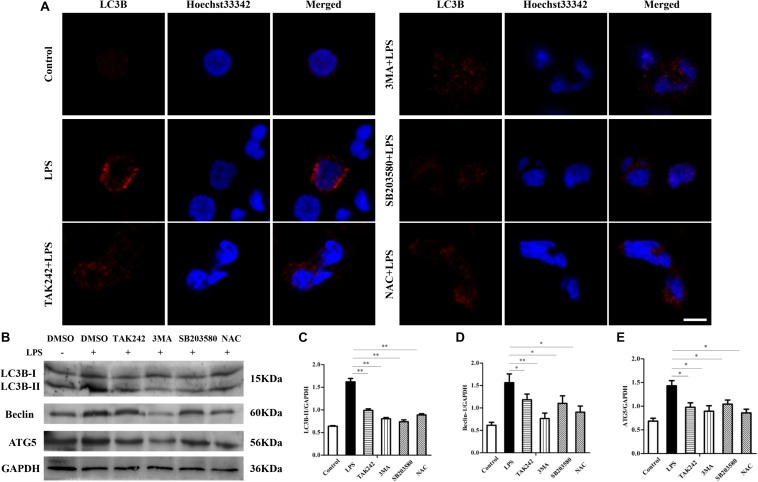
TLR4-induced autophagy in sheep is dependent on the MAPK pathway and the reactive oxygen species. **(A)** Cells were pretreated with either DMSO (0.1%) or TAK242 (30 μM) for 6 h, 3-MA (10 mM) for 6 h, SB203580 (5 μM) for 1 h, or NAC (1 mM) for 2 h, and then the cells were treated with LPS (100 ng/ml) for 6 h. Confocal microscopy was used to analyze the LC3B level. Red: Cy3-labeled LC3B protein; blue, Hoechst33342-labeled nuclei; scale bar: 5 μm. **(B)** The protein level of LC3B, Beclin-1, and Atg5. GAPDH protein was used as a control. **(C–E)** LC3B-II/GAPDH, Beclin-1/GAPDH, and ATG5/GAPDH ratio for each group. ^∗^*P* < 0.05 vs. LPS group; ^∗∗^*P* < 0.01 vs. LPS group.

Although in the data presented above there is a close relationship between TLR4 and LPS-induced autophagy, we still did not know what signaling pathway is involved. As LPS stimulation could activate TLR4-dependent p38 MAPK signaling and ROS, we next surveyed whether these players are involved in the autophagy process. As shown in Figure 6, the TLR4-specific inhibitor TAK242 (30 μM, pretreated for 6 h), the autophagy inhibitor 3-MA (10 mM, pretreated for 6 h), the MAPK-specific inhibitor SB203580 (5 μM, pretreated for 1 h), and the ROS inhibitor NAC (1 mM, pretreated for 2 h) were used independently. The results showed the inhibition of TLR4, autophagy, MAPK, and ROS, and that all led to significantly decreased numbers of autophagosomes under TEM (Figures 6A,B) compared to the LPS group (^∗∗^*P* < 0.01). Similarly, confocal results indicated that the LC3B protein level was also significantly reduced by TAK242, 3-MA, SB203580, and NAC treatment (^∗^*P* < 0.05) (Figure 7A). Furthermore, beclin-1 and ATG5 are both autophagy-related genes. Western blot analysis showed that the inhibited TLR4, autophagy, MAPK signaling, and ROS decreased the protein levels of Beclin-1 and ATG5 (^∗^*P* < 0.05) (Figures 7B–E). In addition, inhibiting TLR4, autophagy, MAPK signaling, and ROS decreased the LC3B-II/GAPDH ratio (^∗^*P* < 0.05). These results suggest that p38 MAPK and ROS were both involved in the TLR4-dependent autophagy process.

### P38 MAPK Signaling and Autophagy Are Involved in Intracellular Oxidative Stress

**FIGURE 8:**
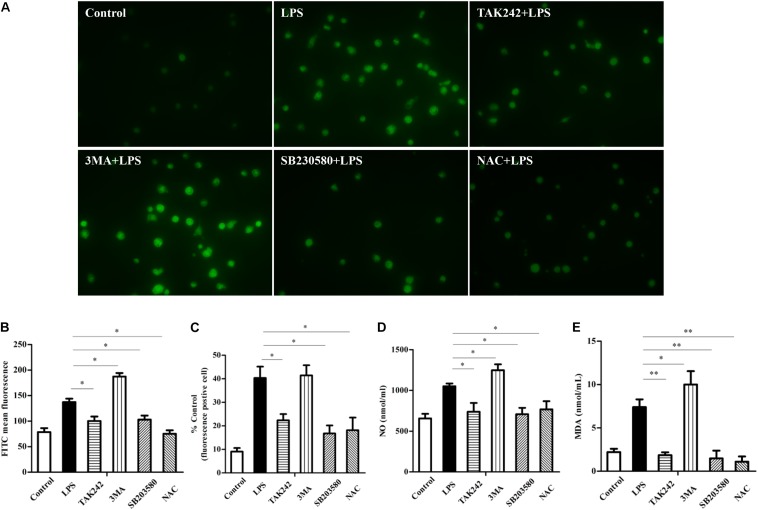
TLR4-induced p38 MAPK signaling and autophagy effect on intracellular oxidative stress. **(A–C)** Analysis of reactive oxygen species (ROS) in sheep. ROS was detected by the DCFH-DA probe and a fluorescence microscope was used to count the positive cells, ×400. **(D)** Level of NO. **(E)** Level of malondialdehyde. All data are presented as the mean ± SEM from three experiments. ^∗^*P* < 0.05 vs. LPS group; ^∗∗^*P* < 0.01 vs. LPS group.

To further explore the relationship between TLR4, autophagy, and oxidation, different inhibitors were used. Figure 8 shows that the inhibition of TLR4 decreased the LPS-induced ROS production and that the levels of NO and MDA also dropped (^∗^*P* < 0.05). After inhibiting autophagy by 3-MA, the ROS, NO, and MDA levels were up-regulated (^∗^*P* < 0.05) compared to the LPS group. In addition, after inhibiting P38 MAPK and ROS, the levels of ROS, NO, and MDA were all lower than those of the LPS group (^∗^*P* < 0.05). The above data suggest that TLR4-associated p38 MAPK signaling and autophagy were involved in regulating intracellular oxidative stress.

### The TLR4-Associated Signaling Pathway, Autophagy, and Oxidative Stress Influence Proinflammatory Cytokine Levels

**FIGURE 9:**
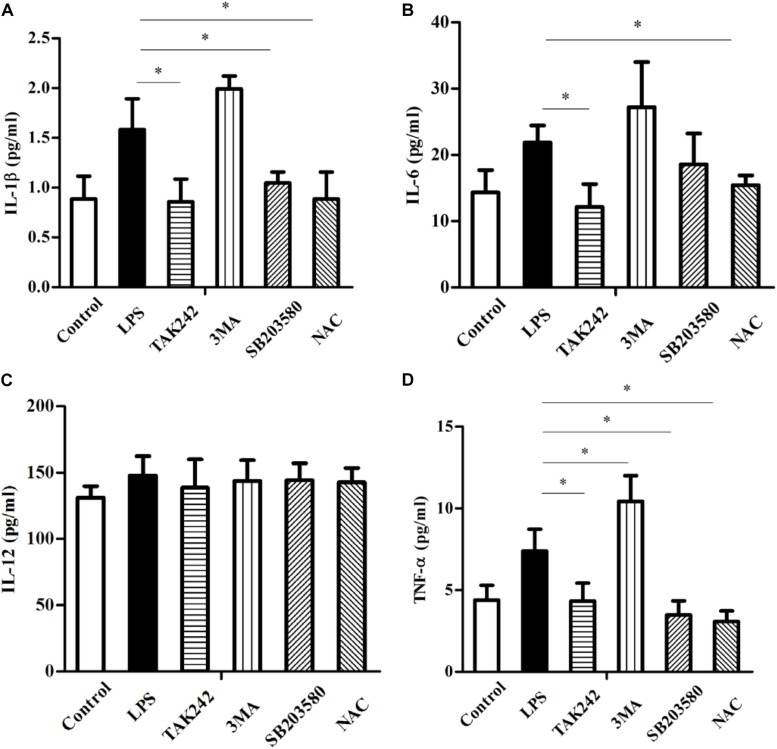
TLR4-induced autophagy and reactive oxygen species effect on proinflammatory cytokine levels (ELISA) in sheep. **(A)** IL-1β levels. **(B)** IL-6 levels. **(C)** IL-12 levels. **(D)** TNF-α levels. All data are presented as the mean ± SEM from three experiments. ^∗^*P* < 0.05 *vs* LPS group.

The LPS-induced activation of TLR4 often leads to the production of multiple cytokines to protect against infection. Figure 9 shows the inhibition of TLR4 decreased the protein levels of IL-1β, IL-6, and TNF-α (^∗^*P* < 0.05). Similarly, the inhibition of the intracellular oxidative stress down-regulated these proteins compared to the LPS group (^∗^*P* < 0.05). Blocking the P38 MAPK resulted in a dramatic drop of IL-1β and TNF-α (^∗^*P* < 0.05) but did not significantly reduce the IL-6 level, whereas 3-MA markedly up-regulated the level of TNF-α (^∗^*P* < 0.05). In addition, although 3-MA increased IL-1β and IL-6, these differences were not statistically significant. Furthermore, all of these manipulations failed to alter the level of IL-12.

### TLR4 Participates in Oxidative Stress and Inflammatory Regulation by Promoting Autophagy *in vivo*

**FIGURE 10:**
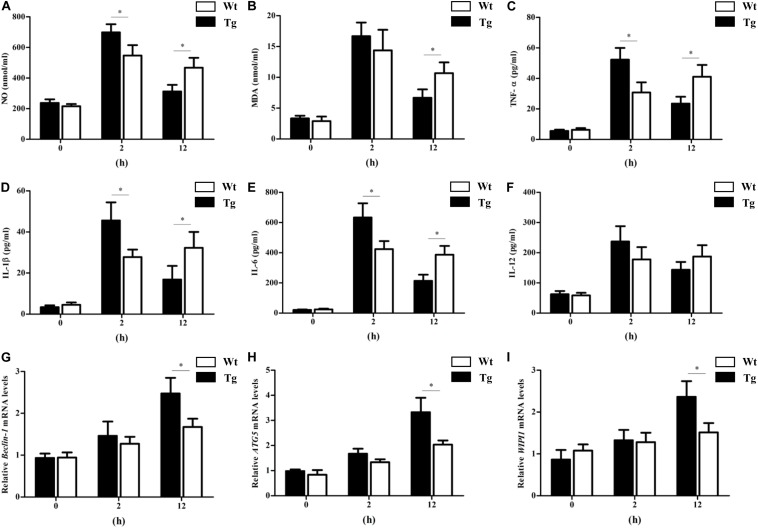
The proinflammatory cytokines, NO and MDA levels, and PBMCs autophagy activity in sheep with LPS treatment *in vivo*. **(A)** NO levels. **(B)** Malondialdehyde levels. **(C)** TNF-α levels. **(D)** IL-1β levels. **(E)** IL-6 levels. **(F)** IL-12 levels. **(G–I)** qRT-PCR analysis of Beclin-1, ATG5, and WIPI1 expression. All data are presented as the mean ± SEM from three experiments. ^∗^*P* < 0.05 vs. Wt group.

To further explore the relationship between TLR4, autophagy, inflammation, and oxidative stress, *in vivo* studies were also performed. The results showed that the transgenic animals had a higher NO level at the early stage (2 h), and then the NO level quickly returned to normal values. The level of NO in transgenic sheep was markedly lower than that in the Wt group at 12 h. The MDA level showed similar trends (Figures 10A,B). In addition, the proinflammatory cytokines IL-1β, IL-6, and TNF-α were significantly increased by LPS challenge in the Tg group at 2 h. By 12 h, the IL-1β, IL-6, and TNF-α levels in the Tg group had dropped dramatically and were significantly lower than those in the Wt group (Figures 10C–E). Furthermore, qRT-PCR tests suggested that the relative mRNA expression of beclin-1, ATG5, and WIPI1 in PBMCs from the Tg group with LPS injection was higher than that in the Wt group at 12 h (Figures 10G–I). The autophagy machinery is initiated under stress conditions to maintain cellular homeostasis, and autophagy is generally thought to inhibit the excessive production of pro-inflammatory cytokines and oxidative stress. These findings suggested that the high levels of TLR4 promoted the PBMC autophagy activity that suppressed the production of proinflammatory cytokines and oxidative stress.

## Discussion

PRRs mainly include TLRs, RIG-I-like receptors, and NOD-like receptors (NLRs) that can recognize most PAMPs. TLRs interact with the myeloid differentiation factor 88 (MyD88) to activate nuclear factor-κB (NF-κB) and MAPKs, which subsequently activate activator protein 1 and induce the production of inflammatory cytokines (Kawai and Akira, 2010; DiDonato et al., 2012; O’Neill et al., 2013). TLR4 interacts with protein CD14 and myeloid differentiation factor 2 to form a complex that is endocytosed into endosomes rapidly (Zanoni et al., 2011). It has been reported that TLR4-mutant cells show lower sensitivity and susceptibility to LPS compared to wild-type cells (Faure et al., 2000; Jilling et al., 2006). In addition, the macrophages of TLR4-deficient mice cannot produce IL-1, IL-6, and IL-12 cytokines under LPS stress (Haynes et al., 2001; Seki et al., 2001). Moreover, mice overexpressing TLR4 showed improved disease resistance (Roy et al., 2006). Our previous study suggested that, compared to controls, the peripheral blood monocytes of Tg sheep overexpressing TLR4 secreted more proinflammatory cytokines, internalized more pathogens, and eliminated pathogens more quickly (Deng et al., 2012; Wang et al., 2018a, b). We also found that the overexpression of TLR4 promoted the activation of both NF-κB and MAPK signaling at an early stage (0–4 h) (Wei et al., 2019). NF-κB and MAPK signaling pathways not only induced the production of pro-inflammatory cytokines but also trigger the release of oxidation intermediates (Ono and Han, 2000; Vladimirov, 2004). Thus, overexpression of TLR4 activated NF-κB and MAPK signaling and was responsible for the increased oxidative stress and production of pro-inflammatory cytokines. Our study showed that inhibiting TLR4 down-regulated the production of IL-1β, IL-6 and TNF-α. Furthermore, inhibition of the p38 MAPK signals downstream of TLR4 decreased the secretion of IL-1β and TNF-α.

Autophagy, as an essential biological pathway with effects on immunity, helps to eliminate intracellular pathogenic microorganisms. Autophagy controls inflammation by interacting with the regulation of the innate immune signals, removing endogenous inflammasome agonists, and affecting immune mediators. Generally, the inhibitors of nuclear factor kappa-B kinase, TAB2/3, and TAK control autophagy together. After PAMP induction, the dissociation of TAB2/3 from Beclin-1 leads to autophagy activation and autophagosome formation (Deretic et al., 2013). The ubiquitination of Beclin-1 by TRAF6 is mediated by TLR4–MyD88 signaling, and then TRAF6 dissociates BCL-2 from Beclin-1. Moreover, it has been reported that TLR4-induced TICAM1 was essential for the activation of autophagy (Zhan et al., 2014). However, cytokine IL-1β may be involved in TRAF 6-dependent Beclin-1 ubiquitination through MyD88 in macrophages (Nakahira et al., 2011; Zhou et al., 2011). A recent report showed that rapamycin (autophagy enhancer) could potently suppress the production of IL-1β, IL-6, TNF-α, and NO in BV2 cells. Furthermore, it has been reported that NF-κB also communicated the connection between autophagy and inflammasome activation. NF-κB promoted the elimination of damaged organelles by the activation of the autophagy receptor SQSTM1 and inhibited IL-1β secretion *via* NLRP3 signals (Zhong et al., 2016). Recently, it was shown that p38 MAPK participates in the production of inflammatory cytokines (Du et al., 2018). These studies suggested that autophagy is closely related to TLR4 and the inflammatory factors. In this study, TLR4 and its downstream signaling p38 MAPK affected the autophagy in sheep monocytes. We found that the overexpression of TLR4 increased the LPS-induced number of autophagosome in monocytes and that the LC3B-II/GAPDH ratio was also increased (Figures 2, 3). Our research further suggests that the down-regulation of TLR4 activity by RNAi (Figure 4) or the TAK242 inhibitor (Figures 6, 7) both decreased the intracellular autophagosomes and the ratio of LC3B-II/GAPDH. In addition, the results also showed that the protein levels of Beclin-1 and ATG5 were down-regulated after TLR4 or p38 MAPK inhibition in Tg peripheral blood monocytes (Figures 6, 7). Previous experiments have mostly focused on the effects of the TLR4-TRAF6 pathway on autophagy (Shi and Kehrl, 2010; Nazio et al., 2013; Zhan et al., 2014). In our Tg animal model, we found that the TLR4–p38 MAPK pathway is also involved in autophagy. The inhibition of TLR4 also decreases the levels of IL-1β, IL-6, and TNF-α, while blocking the p38 MAPK pathway using SB203580 decreases the secretion of IL-1β and TNF-α (Figure 9).

Evidence showed that ROS production is related to TLR4 activation and autophagy (Shin et al., 2013). It has been reported that NF-κB and p38 MAPK signaling involves interactions between autophagy and ROS (He et al., 2013; Zhu et al., 2017). The MAPK pathway mediated by TLR4 under LPS stress participates in cell survival with autophagy (Kim et al., 2013). However, the production of intracellular ROS also plays an essential role in the induction of MAPK, and evidence suggested that ROS could induce MAPK signaling (Bui-Xuan et al., 2010). Moreover, our previous research showed that the MAPK signal is involved in TLR4-mediated bacterial internalization which is mainly about phagocytosis (Wang et al., 2018a). In the current study, the ROS level in sheep over-expressing TLR4 was markedly higher than that in the wild-type group, and it returned to normal by knocking-down TLR4 (Figure 5). Moreover, the suppression of autophagy with 3-MA promoted ROS production and significantly increased the level of NO and MDA (Figure 8). Additionally, the inactivation of p38 MAPK decreased the LPS-induced ROS by p38 MAPK-specific inhibitor SB203580. Similarly, the inactivation of p38 MAPK signals down-regulated the expression of NO and MDA (Figure 8). Evidence suggested that pathogenic Gram-negative bacteria would first stimulate inflammatory secretion and generate ROS which are mostly produced by NOX (Huang et al., 2009). ROS could activate iNOS *via* NF-κB and lead to the release of NO which helped to eliminate pathogenic bacteria by producing peroxidase and superoxide (Heo et al., 2008). The excess oxide would cause organelle damage, but ROS also activates autophagy, which helps to clean the damaged organelles and maintain intracellular homeostasis. Our data in Figure 5B showed that, in the Tg animal, the ROS level increased rapidly at an early stage (peak at 8 h) and then began to fall. Even though the exact underlying mechanisms are unknown, these dynamics may be caused by a high level of autophagy in the Tg group. Taking these data together, under LPS stress, TLR4 induces ROS production to mediate autophagy *via* the p38 MAPK signaling pathway.

To further explore the relationship between TLR4, autophagy, inflammation, and oxidative stress *in vivo*, the transgenic sheep and Wt counterparts were treated with LPS. We found that the serum proinflammatory cytokines levels and oxidative stress in transgenic sheep increased rapidly at an early stage and then began to fall. By 12 h after the LPS challenge, the NO, MDA, and proinflammatory cytokine levels in the transgenic sheep were markedly lower than those in the Wt group. *WIPI1*, *Beclin-1*, and *ATG5* were selected as the indices of autophagy. Because of its reliability, *WIPI1* can be used as a marker of autophagy activity by qRT-PCR analysis (Tsuyuki et al., 2014; Proikas-Cezanne et al., 2015; Tao et al., 2018). *In vivo* experiments show that the autophagy activity of PBMCs increased gradually with LPS treatment, and the autophagy activity in the PBMCs of transgenic sheep was significantly higher than that in the Wt sheep at 12 h after the LPS challenge. These findings *in vivo* were similar to those of the *in vitro* experiments. Cytokines and oxidative stress orchestrate the initiation, development, and recovery during the disease process, and autophagy plays a key role under various stress conditions to maintain cellular homeostasis (Netea-Maier et al., 2016). Several reports showed that TLR4 participated in the regulation of autophagy (Yu et al., 2017; Li et al., 2019). Our previous study found that the overexpression of TLR4 promoted the activation of both NF-κB and MAPK signaling (Wei et al., 2019). In this study, we found that p38 MAPK signaling pathway and ROS production were involved in TLR4-mediated autophagy. Evidence also showed that the autophagy process suppressed all kinds of proinflammatory cytokines and oxidative stress (Kim et al., 2017; Han et al., 2018; Monkkonen and Debnath, 2018). AKT/mTOR signaling is involved in the induction of autophagy to attenuate LPS-induced proinflammatory cytokines. Furthermore, autophagy-deficient ovarian cancer cells display increased ROS and a decrease in autophagy due to the knock-down of BECN1, leading to the up-regulation of ROS and NF-κB activation (Qin et al., 2015; Zhao et al., 2016). So, we thought that the high levels of TLR4 promoted the PBMC autophagy activity that suppressed the production of proinflammatory cytokines and oxidative stress.

In summary, in our current research based on a transgenic sheep model over-expressing TLR4, we surveyed the relationship between ROS, the inflammatory response, and autophagy. The results suggest that transgenic animals have a stronger autophagy activity than the wild-type counterparts *in vitro* and *in vivo*. Furthermore, the autophagy in sheep was mediated by TLR-induced ROS *via* p38 MAPK signaling. Herein we provided a novel animal model to study the effects of TLR4 on autophagy, inflammation, and oxidative stress. In addition, this study also provides valuable insights into Gram-negative bacteriosis.

## Data Availability Statement

All data underlying the findings are included in the article and fully available without restriction.

## Ethics Statement

The animal study was reviewed and approved by the Animal Welfare Committee of the Northeast Agricultural University.

## Author Contributions

Conceptualization was by SW and SD. Data curation and Formal analysis was by SW and XS. Funding acquisition was by ZL and YY. Methodology was by SW. Writing and discussion were carried out by SW, KZ, PJ, MQ, and YY.

## Conflict of Interest

The authors declare that the research was conducted in the absence of any commercial or financial relationships that could be construed as a potential conflict of interest.

## References

[B1] Bui-Xuan N. H. Tang P. M. Wong C. K. Fung K. P. (2010). Photo-activated pheophorbide-a, an active component of *Scutellaria barbata*, enhances apoptosis via the suppression of ERK-mediated autophagy in the estrogen receptor-negative human breast adenocarcinoma cells MDA-MB-231. *J. Ethnopharmacol.* 131 95–103. 10.1016/j.jep.2010.06.007 20558270

[B2] Chen G. Shaw M. H. Kim Y. G. Nunez G. (2009). NOD-like receptors: role in innate immunity and inflammatory disease. *Annu. Rev. Pathol.* 4 365–398. 10.1146/annurev.pathol.4.110807.092239 18928408

[B3] Deng S. Wu Q. Yu K. Zhang Y. Yao Y. Li W. (2012). Changes in the relative inflammatory responses in sheep cells overexpressing of toll-like receptor 4 when stimulated with LPS. *PLoS One* 7:e47118. 10.1371/journal.pone.0047118 23056598 PMC3464238

[B4] Deng S. Yu K. Zhang B. Yao Y. Wang Z. Zhang J. (2015). Toll-Like receptor 4 promotes no synthesis by upregulating GCHI expression under oxidative stress conditions in sheep monocytes/macrophages. *Oxid. Med. Cell Longev.* 2015:359315. 10.1155/2015/359315 26576220 PMC4630417

[B5] Deretic V. Saitoh T. Akira S. (2013). Autophagy in infection, inflammation and immunity. *Nat. Rev. Immunol.* 13 722–737. 10.1038/nri3532 24064518 PMC5340150

[B6] DiDonato J. A. Mercurio F. Karin M. (2012). NF-kappaB and the link between inflammation and cancer. *Immunol. Rev.* 246 379–400. 10.1111/j.1600-065X.2012.01099.x 22435567

[B7] Du K. Zhou M. Li Q. Liu X. Z. (2018). Chlamydia trachomatis inhibits the production of pro-inflammatory cytokines in human PBMCs through induction of IL-10. *J. Med. Microbiol.* 67 240–248. 10.1099/jmm.0.000672 29388547

[B8] Faure E. Equils O. Sieling P. A. Thomas L. Zhang F. X. Kirschning C. J. (2000). Bacterial lipopolysaccharide activates NF-kappaB through toll-like receptor 4 (TLR-4) in cultured human dermal endothelial cells. Differential expression of TLR-4 and TLR-2 in endothelial cells. *J. Biol. Chem.* 275 11058–11063. 10.1074/jbc.275.15.11058 10753909

[B9] Han F. Xiao Q. Q. Peng S. Che X. Y. Jiang L. S. Shao Q. (2018). Atorvastatin ameliorates LPS-induced inflammatory response by autophagy via AKT/mTOR signaling pathway. *J. Cell. Biochem.* 119 1604–1615. 10.1002/jcb.26320 28771872

[B10] Haynes L. M. Moore D. D. Kurt-Jones E. A. Finberg R. W. Anderson L. J. Tripp R. A. (2001). Involvement of toll-like receptor 4 in innate immunity to respiratory syncytial virus. *J. Virol.* 75 10730–10737. 10.1128/JVI.75.22.10730-10737.2001 11602714 PMC114654

[B11] He H. Zang L. H. Feng Y. S. Chen L. X. Kang N. Tashiro S. (2013). Physalin A induces apoptosis via p53-Noxa-mediated ROS generation, and autophagy plays a protective role against apoptosis through p38-NF-kappaB survival pathway in A375-S2 cells. *J. Ethnopharmacol.* 148 544–555. 10.1016/j.jep.2013.04.051 23684722

[B12] Heo S. K. Yun H. J. Noh E. K. Park W. H. Park S. D. (2008). LPS induces inflammatory responses in human aortic vascular smooth muscle cells via Toll-like receptor 4 expression and nitric oxide production. *Immunol. Lett.* 120 57–64. 10.1016/j.imlet.2008.07.002 18675302

[B13] Hoareau L. Bencharif K. Rondeau P. Murumalla R. Ravanan P. Tallet F. (2010). Signaling pathways involved in LPS induced TNFalpha production in human adipocytes. *J. Inflamm.* 7:1. 10.1186/1476-9255-7-1 20148136 PMC2819999

[B14] Huang J. Canadien V. Lam G. Y. Steinberg B. E. Dinauer M. C. Magalhaes M. A. (2009). Activation of antibacterial autophagy by NADPH oxidases. *Proc. Natl. Acad. Sci. U.S.A.* 106 6226–6231. 10.1073/pnas.0811045106 19339495 PMC2664152

[B15] Janeway C. A. Jr. Medzhitov R. (2002). Innate immune recognition. *Annu. Rev. Immunol.* 20 197–216. 10.1146/annurev.immunol.20.083001.084359 11861602

[B16] Jilling T. Simon D. Lu J. Meng F. J. Li D. Schy R. (2006). The roles of bacteria and TLR4 in rat and murine models of necrotizing enterocolitis. *J. Immunol.* 177 3273–3282. 10.4049/jimmunol.177.5.3273 16920968 PMC2697969

[B17] Kawai T. Akira S. (2010). The role of pattern-recognition receptors in innate immunity: update on Toll-like receptors. *Nat. Immunol.* 11 373–384. 10.1038/ni.1863 20404851

[B18] Kim G. D. Oh J. Park H. J. Bae K. Lee S. K. (2013). Magnolol inhibits angiogenesis by regulating ROS-mediated apoptosis and the PI3K/AKT/mTOR signaling pathway in mES/EB-derived endothelial-like cells. *Int. J. Oncol.* 43 600–610. 10.3892/ijo.2013.1959 23708970

[B19] Kim K. Y. Park K. I. Kim S. H. Yu S. N. Park S. G. Kim Y. W. (2017). Inhibition of autophagy promotes salinomycin-induced apoptosis via reactive oxygen species-mediated PI3K/AKT/mTOR and ERK/p38 MAPK-Dependent signaling in human prostate cancer cells. *Int. J. Mol. Sci.* 18:1088. 10.3390/ijms18051088 28524116 PMC5454997

[B20] Li J. Li B. Cheng Y. Meng Q. Wei L. Li W. (2019). The synergistic effect of NOD2 and TLR4 on the activation of autophagy in human submandibular gland inflammation. *J. Oral Pathol. Med.* 48 87–95. 10.1111/jop.12793 30367515

[B21] Monkkonen T. Debnath J. (2018). Inflammatory signaling cascades and autophagy in cancer. *Autophagy* 14 190–198. 10.1080/15548627.2017.1345412 28813180 PMC5902219

[B22] Nakahira K. Haspel J. A. Rathinam V. A. Lee S. J. Dolinay T. Lam H. C. (2011). Autophagy proteins regulate innate immune responses by inhibiting the release of mitochondrial DNA mediated by the NALP3 inflammasome. *Nat. Immunol.* 12 222–230. 10.1038/ni.1980 21151103 PMC3079381

[B23] Nazio F. Strappazzon F. Antonioli M. Bielli P. Cianfanelli V. Bordi M. (2013). mTOR inhibits autophagy by controlling ULK1 ubiquitylation, self-association and function through AMBRA1 and TRAF6. *Nat. Cell Biol.* 15 406–416. 10.1038/ncb2708 23524951

[B24] Netea-Maier R. T. Plantinga T. S. van de Veerdonk F. L. Smit J. W. Netea M. G. (2016). Modulation of inflammation by autophagy: consequences for human disease. *Autophagy* 12 245–260. 10.1080/15548627.2015.1071759 26222012 PMC4836004

[B25] O’Neill L. A. Golenbock D. Bowie A. G. (2013). The history of Toll-like receptors - redefining innate immunity. *Nat. Rev. Immunol.* 13 453–460. 10.1038/nri3446 23681101

[B26] Ono K. Han J. (2000). The p38 signal transduction pathway: activation and function. *Cell. Signal.* 12 1–13. 10.1016/s0898-6568(99)00071-76 10676842

[B27] Pacquelet S. Johnson J. L. Ellis B. A. Brzezinska A. A. Lane W. S. Munafo D. B. (2007). Cross-talk between IRAK-4 and the NADPH oxidase. *Biochem. J.* 403 451–461. 10.1042/BJ20061184 17217339 PMC1876389

[B28] Park H. S. Chun J. N. Jung H. Y. Choi C. Bae Y. S. (2006). Role of NADPH oxidase 4 in lipopolysaccharide-induced proinflammatory responses by human aortic endothelial cells. *Cardiovasc. Res.* 72 447–455. 10.1016/j.cardiores.2006.09.012 17064675

[B29] Proikas-Cezanne T. Takacs Z. Donnes P. Kohlbacher O. (2015). WIPI proteins: essential PtdIns3P effectors at the nascent autophagosome. *J. Cell Sci.* 128 207–217. 10.1242/jcs.146258 25568150

[B30] Qin W. Li C. Zheng W. Guo Q. Zhang Y. Kang M. (2015). Inhibition of autophagy promotes metastasis and glycolysis by inducing ROS in gastric cancer cells. *Oncotarget* 6 39839–39854. 10.18632/oncotarget.5674 26497999 PMC4741864

[B31] Roy M. F. Lariviere L. Wilkinson R. Tam M. Stevenson M. M. Malo D. (2006). Incremental expression of Tlr4 correlates with mouse resistance to *Salmonella* infection and fine regulation of relevant immune genes. *Genes Immun.* 7 372–383. 10.1038/sj.gene.6364309 16738669

[B32] Ryan K. A. Smith M. F. Jr. Sanders M. K. Ernst P. B. (2004). Reactive oxygen and nitrogen species differentially regulate Toll-like receptor 4-mediated activation of NF-kappa B and interleukin-8 expression. *Infect. Immun.* 72 2123–2130. 10.1128/iai.72.4.2123-2130.2004 15039334 PMC375203

[B33] Sciarretta S. Volpe M. Sadoshima J. (2014). NOX4 regulates autophagy during energy deprivation. *Autophagy* 10 699–701. 10.4161/auto.27955 24492492 PMC4091158

[B34] Seki E. Tsutsui H. Nakano H. Tsuji N. Hoshino K. Adachi O. (2001). Lipopolysaccharide-induced IL-18 secretion from murine Kupffer cells independently of myeloid differentiation factor 88 that is critically involved in induction of production of IL-12 and IL-1beta. *J. Immunol.* 166 2651–2657. 10.4049/jimmunol.166.4.2651 11160328

[B35] Shi C. S. Kehrl J. H. (2010). TRAF6 and A20 regulate lysine 63-linked ubiquitination of Beclin-1 to control TLR4-induced autophagy. *Sci. Signal.* 3:ra42. 10.1126/scisignal.2000751 20501938 PMC6335036

[B36] Shin S. Jing K. Jeong S. Kim N. Song K. S. Heo J. Y. (2013). The omega-3 polyunsaturated fatty acid DHA induces simultaneous apoptosis and autophagy via mitochondrial ROS-mediated Akt-mTOR signaling in prostate cancer cells expressing mutant p53. *Biomed. Res. Int.* 2013:568671. 10.1155/2013/568671 23841076 PMC3691929

[B37] Singh R. Cuervo A. M. (2011). Autophagy in the cellular energetic balance. *Cell Metab.* 13 495–504. 10.1016/j.cmet.2011.04.004 21531332 PMC3099265

[B38] Suzuki Y. Hattori K. Hamanaka J. Murase T. Egashira Y. Mishiro K. (2012). Pharmacological inhibition of TLR4-NOX4 signal protects against neuronal death in transient focal ischemia. *Sci. Rep.* 2:896. 10.1038/srep00896 23193438 PMC3508453

[B39] Takeuchi O. Akira S. (2010). Pattern recognition receptors and inflammation. *Cell* 140 805–820. 10.1016/j.cell.2010.01.022 20303872

[B40] Tao J. Yang M. Wu H. Ma T. He C. Chai M. (2018). Effects of AANAT overexpression on the inflammatory responses and autophagy activity in the cellular and transgenic animal levels. *Autophagy* 14 1850–1869. 10.1080/15548627.2018.1490852 29985091 PMC6152527

[B41] Tse K. H. Chow K. B. Leung W. K. Wong Y. H. Wise H. (2014). Lipopolysaccharide differentially modulates expression of cytokines and cyclooxygenases in dorsal root ganglion cells via Toll-like receptor-4 dependent pathways. *Neuroscience* 267 241–251. 10.1016/j.neuroscience.2014.02.041 24607321

[B42] Tsuyuki S. Takabayashi M. Kawazu M. Kudo K. Watanabe A. Nagata Y. (2014). Detection of WIPI1 mRNA as an indicator of autophagosome formation. *Autophagy* 10 497–513. 10.4161/auto.27419 24384561 PMC4077887

[B43] Vladimirov Y. A. (2004). Reactive oxygen and nitrogen species: diagnostic, preventive and therapeutic values. *Biochemistry* 69 1–3. 10.1023/b:biry.0000016343.21774.c4 15127743

[B44] Wang S. Cao Y. Deng S. Jiang X. Wang J. Zhang X. (2018a). Overexpression of toll-like receptor 4-linked mitogen-activated protein kinase signaling contributes to internalization of *Escherichia coli* in sheep. *Int. J. Biol. Sci.* 14 1022–1032. 10.7150/ijbs.25275 29989103 PMC6036738

[B45] Wang S. Deng S. Cao Y. Zhang R. Wang Z. Jiang X. (2018b). Overexpression of toll-like receptor 4 contributes to phagocytosis of *Salmonella Enterica* serovar typhimurium via phosphoinositide 3-Kinase signaling in sheep. *Cell Physiol. Biochem.* 49 662–677. 10.1159/000493032 30165358

[B46] Wei S. Yang D. Yang J. Zhang X. Zhang J. Fu J. (2019). Overexpression of Toll-like receptor 4 enhances LPS-induced inflammatory response and inhibits *Salmonella* Typhimurium growth in ovine macrophages. *Eur. J. Cell Biol.* 98 36–50. 10.1016/j.ejcb.2018.11.004 30522781

[B47] Yu S. X. Du C. T. Chen W. Lei Q. Q. Li N. Qi S. (2015). Genipin inhibits NLRP3 and NLRC4 inflammasome activation via autophagy suppression. *Sci. Rep.* 5:17935. 10.1038/srep17935 26659006 PMC4675967

[B48] Yu T. Guo F. Yu Y. Sun T. Ma D. Han J. (2017). *Fusobacterium nucleatum* promotes chemoresistance to colorectal cancer by modulating autophagy. *Cell* 170 548–563.e16. 10.1016/j.cell.2017.07.008 28753429 PMC5767127

[B49] Zanoni I. Ostuni R. Marek L. R. Barresi S. Barbalat R. Barton G. M. (2011). CD14 controls the LPS-induced endocytosis of toll-like receptor 4. *Cell* 147 868–880. 10.1016/j.cell.2011.09.051 22078883 PMC3217211

[B50] Zhan Z. Xie X. Cao H. Zhou X. Zhang X. D. Fan H. (2014). Autophagy facilitates TLR4- and TLR3-triggered migration and invasion of lung cancer cells through the promotion of TRAF6 ubiquitination. *Autophagy* 10 257–268. 10.4161/auto.27162 24321786 PMC5396095

[B51] Zhao Z. Zhao J. Xue J. Zhao X. Liu P. (2016). Autophagy inhibition promotes epithelial-mesenchymal transition through ROS/HO-1 pathway in ovarian cancer cells. *Am. J. Cancer Res.* 6 2162–2177. 27822409 PMC5088283

[B52] Zhong Z. Umemura A. Sanchez-Lopez E. Liang S. Shalapour S. Wong J. (2016). NF-kappaB restricts inflammasome activation via elimination of damaged mitochondria. *Cell* 164 896–910. 10.1016/j.cell.2015.12.057 26919428 PMC4769378

[B53] Zhou R. Yazdi A. S. Menu P. Tschopp J. (2011). A role for mitochondria in NLRP3 inflammasome activation. *Nature* 469 221–225. 10.1038/nature09663 21124315

[B54] Zhu J. Yu W. Liu B. Wang Y. Shao J. Wang J. (2017). Escin induces caspase-dependent apoptosis and autophagy through the ROS/p38 MAPK signalling pathway in human osteosarcoma cells in vitro and in vivo. *Cell Death Dis.* 8:e3113. 10.1038/cddis.2017.488 29022891 PMC5682655

